# User Perception of a Smartphone App to Promote Physical Activity Through Active Transportation: Inductive Qualitative Content Analysis Within the Smart City Active Mobile Phone Intervention (SCAMPI) Study

**DOI:** 10.2196/19380

**Published:** 2020-08-05

**Authors:** Anna-Karin Lindqvist, Stina Rutberg, Emmie Söderström, Anna Ek, Christina Alexandrou, Ralph Maddison, Marie Löf

**Affiliations:** 1 Division of Health, Medicine and Rehabilitation Department of Health Sciences Luleå University of Technology Luleå Sweden; 2 Department of Health, Medical and Caring Sciences Linköping University Linköping Sweden; 3 Department of Clinical Science Intervention and Technology Karolinska Institutet Huddinge Sweden; 4 Institute for Physical Activity and Nutrition School of Exercise and Nutrition Sciences Deakin University Melbourne Australia; 5 Department of Biosciences and Nutrition Karolinska Institutet Huddinge Sweden

**Keywords:** behavior change, smartphone intervention, physical activity, user perception, active transportation, mobile app, inductive qualitative content analysis, mobile health, social cognitive theory, mHealth

## Abstract

**Background:**

Physical inactivity is globally recognized as a major risk factor for morbidity, particularly the incidence of noncommunicable diseases. Increasing physical activity (PA) is therefore a public health priority. Engaging in active transportation (AT) is a viable approach for promoting daily PA levels. Mobile health interventions enable the promotion of AT to a larger population. The Smart City Active Mobile Phone Intervention (SCAMPI) study was a randomized controlled trial designed to evaluate the ability of a behavior change program delivered via a smartphone app to motivate participants to increase their PA by engaging in AT.

**Objective:**

This qualitative study aims to examine the acceptance and user experience of the app promoting AT that was used in the SCAMPI trial (the TRavelVU Plus app).

**Methods:**

A total of 17 residents of Stockholm County (13 women; age range 25-61 years) who completed the 3-month app-based behavioral change program (delivered through the TRavelVU Plus app) in the SCAMPI randomized controlled trial during 2018 agreed to participate in a semistructured telephone-based interview. These participants were well representative of the whole intervention group (n=127) in terms of baseline characteristics such as age, sex, and area of residence. The interviews were audiorecorded, transcribed verbatim, and analyzed using an inductive qualitative content analysis.

**Results:**

The content analysis revealed 2 themes and 4 subcategories. The first theme, “main motivators: monitoring and messages,” highlighted that monitoring AT and being able to set weekly goals using the app were the primary motivators reported by study participants. The second theme, “acceptable but modifiable,” reflects that the app was well accepted and effectively encouraged many participants to use more AT. Nevertheless, there were functions in the app that require modification. For example, while the semiautomated travel tracking feature was appreciated, participants found it time-consuming and unreliable at times.

**Conclusions:**

This study contributes novel insight into adults’ experiences of using a mobile app to promote the use of AT. The results showed that the app was well accepted and that self-monitoring and goal setting were the main motivators to engage in more AT. The semiautomated tracking of AT was appreciated; however, it was also reported to be energy- and time-consuming when it failed to work. Thus, this feature should be improved going forward.

**Trial Registration:**

ClinicalTrials.gov NCT03086837; https://clinicaltrials.gov/ct2/show/NCT03086837

**International Registered Report Identifier (IRRID):**

RR2-10.1186/s12889-018-5658-4

## Introduction

Over 30% of the global population is physically inactive [1]. This is alarming, as physical inactivity is one of the greatest risk factors for mortality worldwide [2] and contributes to the development of most noncommunicable diseases [3]. Therefore, promoting physical activity (PA) is a public health priority [4]. In Sweden, there has been a pronounced decline in mean cardiorespiratory fitness in adults between 1995 and 2017, and the proportion of people with low cardiorespiratory fitness has almost doubled [5]. One factor potentially associated with this fitness loss is preferred mode of transportation; over the past two decades, the use of active transportation (AT) has decreased while motorized transportation use has increased [6]. Municipalities over the world have tried different strategies, including banning cars from their city centers and redesigning urban streets in order to increase walking and cycling to ensure individuals can transport themselves actively and become healthier [7]. As health benefits can occur even with modest improvements in PA levels [8], engaging in AT is an effective, impactful way to increase the total amount of PA [3,9]. Moreover, AT has been inversely associated with noncommunicable diseases, including obesity, type 2 diabetes, cardiovascular disease, and falls [10-12]. However, changing people’s behaviors toward AT is especially challenging because external factors influence travel mode, such as convenience and weather conditions [13]. This is particularly true in Sweden and other Nordic countries, where winters are long, cold, dark, and snowy [14]. Thus, new and innovative approaches to changing the attitudes and behaviors regarding AT are needed. In this project, AT was defined as walking or biking parts of or the entire way to a destination.

The extensive development of information and communication technologies characteristic of modern society enables interventions to reach large populations [15]. The benefits of a mobile health (mHealth)–based intervention are that it can be delivered at scale and at any time, can be tailored to meet peoples´ needs, and is more cost-effective than face-to-face counseling [16]. Specifically, mHealth programs have already proven to be useful in promoting PA and weight loss [17,18].

Despite their utility, it is important to understand how people perceive and use mHealth interventions in order to improving the effectiveness, engagement, and acceptance of existing and future mHealth apps. Increasingly, studies are now including an assessment of user perceptions. For example, Dennison et al explored user perceptions of mobile apps for behavior change among healthy young adults and showed that users expected apps to be accurate, legitimate, secure, and able to record and track behavior and goals [19]. Participants also expected health apps to require minimal effort to operate [19]. Another study examined the design and content elements of health apps that facilitate or impede usage from the user perspective [20]. Their findings are largely similar to Dennison et al [19], with the addition of participants requesting individually tailored information to meet their needs. Additionally, two studies described user perceptions of mobile apps in the health care sector, one for medication management among older adults [21] and one regarding the acceptability of an app for diabetes self-management [22]. Through assessing users’ perceptions of mHealth apps, it is possible to improve the design of future mHealth interventions that are effective and accepted by end users [20]. To date, no study that we are aware of has explored user perceptions of an app aiming to promote AT.

Recently, the main results from the Smart City Active Mobile Phone Intervention (SCAMPI) randomized controlled trial (ClinicalTrials.gov NCT03086837) [23] were published [24]. The SCAMPI trial aimed to evaluate the effectiveness of a 3-month behavior change program delivered through a mobile phone app to promote AT (TRavelVU Plus app) on moderate-to-vigorous PA. The results showed that there was a moderate effect of the intervention on moderate-to-vigorous PA at 6 months after baseline, which corresponded to around 30% of the weekly recommendation [24]. In this qualitative study, we examine the acceptance and user experience of the app used to promote AT in the SCAMPI trial (ie, the TRavelVU Plus app).

## Methods

### Study Design and Recruitment

The SCAMPI trial was conducted between September 2017 and September 2018 in Stockholm County, Sweden (N=254) [23]. Full details on the trial’s design and methods, as well as the main intervention results, are published elsewhere [23,24]. Briefly, all participants downloaded the TRavelVU app and used it for baseline and follow-up measurements of their AT. The TRavelVU app automatically registered participants’ use of transportation (time, duration, and mode) using GPS coordinates. In the evening, participants were asked to review and, if necessary, manually revise travel and locations in the app (ie, the recordings of travel were semiautomated). Travel behaviors were presented graphically for daily, weekly, and monthly AT use. After baseline measurements, participants were randomized into either the control (n=127) or intervention group (n=127). Participants allocated to the intervention group received the TRavelVU Plus app, which included a behavior change program grounded in Bandura’s social cognitive theory and the principles of social ecology [25,26]. The behavior change program comprised a weekly goal-setting function and feedback on the weekly goal to inspire additional AT. Messages also provided encouragement to engage in AT and offered strategies to do so. Detailed descriptions of the TRavelVU and the TRavelVU Plus apps are available in the study protocol [23].

After completing the intervention, participants in the intervention group were contacted by email and asked to participate in a semistructured telephone-based interview regarding their experiences using the app. Participants received information about the purpose of the interview and were informed that participation was voluntary. In total, 17 participants agreed to participate, and a telephone interview was scheduled with each. The 17 participants interviewed were representative of the intervention group, with the majority of them being women (13/17, 76%), having a mean age of 51 (SD 11) years, having a university degree (10/17, 59%), and spending on average 59 (SD 21) minutes per day in moderate-to-vigorous PA. None of these results were statistically different (*P* values ranging from .16 to .89) from the corresponding results for the entire intervention group (n=127), of which the majority of participants were women (78/127, 61.4%), with a mean age of 47 (SD 11), a university degree (80/127, 63.0%), and a mean moderate-to-vigorous PA of 60 (SD 28) minutes per day. Furthermore, the entire intervention group and the participants in the interviews lived in similar residential areas (urban) of Stockholm County. At baseline, there was a wide range in their use of active transportation, as reported previously [24], and no difference between the entire group and the participants in this qualitative study (intervention group: n=127; mean AT 58 min/day, SD 29; qualitative study: n=17; mean AT 55 min/day, SD 22; *P*=.68). Informed consent was obtained at the start of the interview. The study and the consent procedures were approved by the regional ethical board in Stockholm (January 11, 2017: dnr 2016/2403-31 and March 22, 2018: dnr 2018/615-32).

### Data Collection

To explore the study participants´ perceptions of the TRavelVU Plus app, a semistructured interview guide was developed. This guide included questions regarding the app’s layout and function, as well as the feasibility and acceptability of using the app. We explicitly asked whether participants had experienced problems with the app and if they had suggestions for improvements. To maximize the amount and depth of the data collected in the interview, follow-up questions were posed, such as “Could you tell me more?” The interviews lasted between 16 and 30 minutes, were audiorecorded, and were transcribed verbatim. ES, a research assistant, conducted all interviews.

### Data Analysis

The interviews were analyzed using an inductive qualitative content analysis inspired by Graneheim and Lundman [27]. Initially, the transcribed interviews were carefully read several times by A-KL and SR for greater understanding of the material. The text was then divided into meaning units guided by the aim of the study. The meaning units were coded close to the original text. The codes were compared, contrasted, and sorted into preliminary subcategories and themes. After rereading the interviews, to verify that no important information had been omitted, 2 themes containing 2 subcategories were created. A-KL and SR have extensive experience in qualitative research, and they were primarily responsible for the analysis; however, all authors contributed to the final result. The authors have different backgrounds and areas of expertise, such as physiotherapy (A-KL, SR), physical activity and public health nutrition (ES, CA, ML), and clinical nutrition (AE). This variety of experiences and perspectives increases the likelihood of providing a more nuanced interpretation of the results [28]. In accordance with Graneheim and Lundman [27], quotations were included to strengthen the credibility of the study.

## Results

The analysis resulted in 2 themes with 2 subcategories each to describe participants’ perceptions of using the TRavelVU Plus app.

### Main Motivators: Monitoring and Messages

Monitoring AT and setting weekly goals in the app were perceived to be the main motivators to use AT, for example, by choosing transportation options other than a car. A majority of participants also reported being motivated by the daily messages, which served as reminders to engage in AT. However, at the time of the interview, they could not always remember the actual content of the messages.

#### Going for Goals

Two of the most appreciated features of the app were the self-monitoring function and the ability to set a weekly AT goal. Participants highlighted how goal setting served as a motivator for behavioral change or for maintaining their current level of AT. Some participants set goals that they could easily reach and, therefore, did not become more active than before. In contrast, others challenged themselves and strived to constantly improve their behavior. When the set goal became within reach, it especially triggered some participants to use more AT to ensure that the goal was achieved. For participants who had not set challenging enough goals or fell too far behind with no chance of recovering, the goal-setting function was not perceived to be motivating. A feedback message was sent to participants who had not reached their goal by the end of the week. Some participants reported that this feedback on the set goal was provided too late and suggested that, instead, messages should be sent earlier in the week; then the messages would motivate participants to reach their goal, as it was seen by some participants as a competition against themselves. In addition, some participants suggested including a platform for connecting with other app users, sharing their progress, and maybe even competing against each other.

The weekly goal setting affected me a lot since it made me more physically active and gave me a sense of pride. I made sure to reach my goal, especially if I was very close to achieving it.

Woman, 45 years old, interview 6

Receiving feedback and getting graphical statistics on their transportation mode increased participants’ awareness of their behavior and inspired several participants to make better decisions and use AT more often. For example, participants reported that they checked the map of the route they had walked during the day and reflected on possible longer or shorter routes. Additionally, the graphics were used to see whether they could have saved time or somehow been more physically active. Even though some reported that the app made them more active, especially through promoting AT despite poor weather or other discouraging circumstances, many said that the app had little or no effect on their PA level. These participants were usually highly active prior to using the app or affected by external factors, such as large quantities of snow or slippery roads during the intervention, long transportation distances that obviate the use of AT, or practical things, such as dropping children off at preschool.

I think it is good to become more aware of my modes of transportation … If I walk about 10,000 steps a day, then I am satisfied, but if I also see that I take the car for 5 minutes, 10 times a day, then I realize that I could have walked even more. It provides more insights about how you travel during a day.

Woman, 25 years old, interview 3

#### Messages: Encouraging or Just a Frustration?

The app posted encouraging messages, to which participants had varying responses. Some expressed that messages affected their transportation choices and, therefore, found them useful. Participants with intentions to change their behavior also viewed the messages as helpful and effective reminders to engage in more AT, for example, by getting off the bus earlier and walking the remaining part of the way to their destination. They found that the messages prevented them from getting stuck in habits and forgetting about other alternatives.

In contrast, other participants reported that the messages made them feel bad for not using AT or not being physically active. Some participants found the messages to be somewhat annoying, especially when messages were similar or repeated. Others appreciated that the messages had a positive tone. Practical messages, for example, with information on how to take care of your bicycle, were brought up as helpful. Participants also said that it was motivating to receive facts on the health-related aspects of PA. One participant was especially inspired by a message about sedentary behavior that included information on the importance of breaking up sedentary portions of the day and how one could spread PA throughout the day rather than doing it all at once (eg, in the evening).

One of the most useful things was the messages that contained inspiration and suggestions on how to change behavior. Perhaps not the most fun, but the most useful, because then I realized that I have not done any active travel today. It made me think, perhaps I should get off the bus earlier and walk the rest of the way.

Woman, 61 years old, interview 7

### Acceptable but Modifiable

Taken together, the interviews indicated that the app was generally well accepted by the participants and that it had encouraged many to use more AT and public transportation. The participants graded the app overall an average of 3.5 on a scale from 0 to 5 (range 2.0-4.5). Nevertheless, the participants also identified several issues and areas for improvement.

#### Semiautomated Registration: Heaven and Hell

Most participants appreciated the app’s semiautomated registration of their transportation activity. However, registration was also the feature with the most reported problems. The app sometimes erroneously changed the mode of transportation, for example, when passing by a subway station. Additionally, transportation would sometimes incorrectly change modes, for example, from driving to biking, if the velocity suddenly changed from a relatively high speed to a lower one (eg, due to traffic). Some participants said it sometimes felt as if the app was just guessing their mode of transportation. They expressed that occasionally it would register a route as a straight line between A and B, which was probably due to the lack of GPS points in some areas. Resolving these issues by correcting the route and mode of transportation required a lot of work, according to the participants. One of the participants did not appreciate that the app could track your whereabouts and therefore deleted it as soon as the intervention was over. Participants expressed different experiences with respect to the workload required to manually change the registered data in the app. Some participants expressed it was difficult and time-consuming, while others found it annoying but manageable. Some participants reported that having to correct the registration became boring and that the corrections were, at times, less accurate. The app’s ability to automatically choose an activity connected to a certain place was considered both a strength and a weakness. For example, if the same activity was always performed at one place, the feature was helpful, but when visiting a place where several activities could be performed, this default function required participants to spend more time on corrections than it would have taken them to register the activity themselves.

The app gave me some trouble and sometimes it was difficult to use. It has been random, sometimes it made correct assumptions and sometimes not. Sometimes it took 15 minutes to correct registrations and sometimes it just worked. Sometimes it says that I do not have a network connection even though I do, and without it the app does not work.

Man, 63 years old, interview 2

I think the app learned how to register my travels well. You did not need to do everything all over again every time, except maybe change the travel mode to bus instead of bike, but that was really easy to do.

Man, 56 years old, interview 8

#### Tailored Transportation

To increase the effectiveness of the app, many participants suggested that it should be more tailored to the individual to better address their specific needs and circumstances. Some participants wanted the app to not register travel modes that they never used or were not able to use (eg, not register a train ride if that was never an option in the area). One participant suggested that displaying the most used travel modes and the most frequently visited places at the top of the list in the app would make it more user-friendly. Some also suggested hiding options that were never used (eg, remove motorbike as an option if the participant did not own one). Another example was more individually tailored messages. Some participants received messages about taking the subway even though it was not a travel option in their area or about riding a bike when they had no access to one. Some messages included season-specific information not suitable all year, which was highlighted specifically by many as relevant to improve. Some expressed that they wanted to turn off the messages and instead use the app for an overview of their travels (ie, time use statistics). One participant requested messages about traffic conditions, for example, when the subway was not working or a traffic accident had occurred, or real-time route optimization information, such as “If you get off the bus now you will reach your destination sooner.” Other participants requested more startling information to catch their attention, like how health is affected by a mere 20 minutes of AT.

I would have graded the app higher if it was possible to adjust it to my preferences. I do not bike, and I have received a lot of information about cycling, and I do not take the bus, but the app often suggested that I travelled by bus when it registered my travels.

Man, 47 years old, interview 1

Some participants stated that they might have used the app more frequently if the statistics displayed were more appealing to them and if the app were more interactive. For example, many expressed that the app would have been more informative if it included measurement of total PA, since much of their activity never got included in the statistics, and some participants wanted to see their calorie expenditure as well. Participants also suggested connecting the TRavelVU Plus app to other apps, like Runkeeper (Asics Corp) or Moves (Facebook Inc), to summarize the amount of total PA. Some indicated that the app would benefit from a different start screen (eg, one that displays how much of a participant’s goal remains). Some participants had trouble finding the feature used to set weekly goals if they did not do it shortly after receiving the descriptive message with a link to the goal-setting function. Along the same lines, some participants found only being able to set a goal for the upcoming week on Sundays and no later to be limiting. Moreover, several participants reported wishing that they had set more goals than they actually did and suggested incorporating a function that forced you to choose whether you wanted to set a goal or not for that week, which thereby served as a reminder to set a goal, ideally in a timely fashion.

I would have liked an app that includes a wide variety of health measurements. Now there are apps for movement and apps for eating, but if you got them all in one app I would use it a lot more. If the app included other health components, I could have set goals that were more attractive to me.

Woman, 25 years old, interview 3

## Discussion

This study explored healthy adults’ perceptions of using a smartphone app developed to promote the use of AT as a means to increase daily PA.

### Principal Findings

Overall, results showed that the app was perceived as acceptable by study participants and was able to motivate them to choose active rather than motorized forms of transportation. Similar to the results published by Peng et al [20], the participants appreciated the semiautomated collection of AT data. However, as this function sometimes failed, participants had to make corrections in the app, and this was perceived as unnecessarily time-consuming and caused undue frustration. In this context, it is relevant to note that Dennison et al [19] found that self-monitoring, goal setting, and receiving feedback were important features, as long as the input effort required was not perceived as too burdensome. The participants in that study reported that automated, accurate, and detailed data registration programs were still highly needed. Indeed, a high-detail feature for the automatic recognition of AT was included in this study. However, our results indicated that some refinement is required to improve the accuracy level, which is an important topic for future research.

Understanding the mechanisms underlying health behavior changes inspired by a mobile app is relevant to connecting to the theories that the intervention is grounded in and the behavior change techniques included. Hoj et al [29] describe an association between self-reported app engagement and impact on the theory-based mechanism of behavior change. Our results indicate that self-monitoring their transportation mode increased participants’ awareness of their behavior. This is consistent with the transtheoretical model, which describes health behavior change as occurring through multiple stages, of which awareness is the first stage [30]. Self-monitoring is also one of 3 steps in the behavior change process, according to Bandura’s social cognitive theory [31]. This is important, as the quantitative outcome of this project showed an increase in moderate-to-vigorous PA at the 6-month follow-up [24]. By creating awareness, the app might have initiated a process of behavior change that was first seen after 6 months.

According to the participants, the most motivating features of the app were goal setting and self-monitoring, which correspond to the second step, judgment, in Bandura’s [31] theory. Goal setting and self-monitoring are valuable in supporting and sustaining health behavior changes using mobile app interventions [19,20]. Our study adds to the evidence of the importance of putting extra effort into reaching a goal; however, some participants also reported losing motivation if they perceived the goal to be unreachable or easily achievable without changing their behavior. This is comparable to the results from a recent meta-analysis that concluded that goal setting robustly affects behavioral changes when the goal is specific and challenging enough [32]. The authors [32] also emphasized that goal setting is favored at the group level, where it is set and monitored publicly (preferably by another person). Along this line of thought, the participants in our study suggested that the app should allow and encourage connections between its users, a feature that could promote behavior change, as someone else is also monitoring the progress. Thus, in summary, the goal-setting and self-monitoring features should be kept in future versions of the TRavelVU Plus app. In addition, a feature that allows participants the option to share their goals and achievements with other participants could be added.

The SCAMPI study aimed to promote AT among healthy adults to increase their PA. However, participants reported that they might have used the app more frequently if it had included statistics concerning their general health or all their types of PA (including physical exercise). This could be important to consider when designing behavior change interventions with a focus on AT. Goal setting is important when changing behavior, and the goals must correspond to participants’ expectations [32]. Otherwise, it might affect both the use and the perception of the app. Thus, for future versions of the TRavelVU app, as well as for other similar tools, the ability to monitor other types of PA (eg, gym classes, running) should be considered as an additional feature.

In the present study, participants who intended to increase their PA perceived messages as motivating, as they reminded them to use more AT. This is in line with Prestwich et al [33], who described increased PA in participants who intended to change their behavior and received motivating text messages compared with a control group that did not receive reminders or did not have any intention of changing their behavior. Also, McDermott et al [34] found that combining self-monitoring with other behavior change techniques, such as information, more effectively promotes PA and healthy eating than interventions based on self-monitoring alone. Self-monitoring and reminders are valued features and possibly explain the results in Karppinen et al’s [35] study on healthy habit formation. The participants in our study called for more tailored information in the messages promoting AT, as unnecessary information only irritated them. This is similar to the results published by Peng et al [20], which concluded that participants requested more personalized information. In addition, Peng et al [20] noted that the inclusion of tailored information and increased personalization can encroach on privacy protection issues and feel invasive to some users. This was also observed in our study, as one participant expressed concerns about the app collecting information on his travel habits and uninstalled the app as soon as the intervention was over, while others explicitly recommended including more personalized information. Maintaining the delicate balance between personalization and anonymity is a challenge facing the entire field of mHealth. With increasing numbers of commercially available apps for PA entering the market, users are required to share more personal and contextual data (eg, geolocation), which makes the balance between personalization and anonymity problematic. More research is required to understand how best to manage this balance.

### Strengths and Limitations

Qualitative evaluations of novel intervention methods are critical, as user experience and acceptance are key in ensuring widespread adoption and implementation and in guiding the design of future interventions. Here we present a qualitative evaluation of the SCAMPI trial that further explains and complements the quantitative main findings of the study [24]. Given that participation in this follow-up study was voluntary, it is possible that the participants willing to be interviewed had a more positive attitude toward the project and the app than those who were unwilling to participate further by being interviewed. Therefore, we asked them to highlight problems and identify areas for improvement in the app, which allowed us to paint a more nuanced picture of their perceptions. As reported in our main outcome paper [24], a major strength of the SCAMPI trial was that participants were invited from a random sample drawn by Statistics Sweden. Nevertheless, as is common in research, participants in the entire intervention group (n=127) and participants in this qualitative evaluation (n=17) had higher educational attainment (10/17, 59% had a university degree) than the nonresponders. Moreover, participants were already active prior to joining the study, spending on average 59 (SD 21) minutes per day in moderate-to-vigorous PA; this level of interest in PA may have affected the result of this study. For instance, it is possible that individuals already engaging in PA may also be users of activity apps. Thus, future studies should also include populations that are more sedentary. Finally, this study was limited by having only 17 out of the 127 original participants participate in the interviews. However, we ensured that this subpopulation was representative of the original group and found that a sample of this size was sufficient to generate interviews that contained a rich, broad variety of experiences.

### Conclusions

This article contributes novel information about healthy adults’ experiences using an app that promotes AT as a means toward increased PA. The results showed that the app was acceptable and that participants who were ready to make a behavior change were motivated by self-monitoring, goal setting, and receiving reminder messages to use AT. In addition, all participants reported that the app increased their awareness of their travel habits, which is the initial step required for behavior change to occur. Taken together, our results showed that the app’s features were appreciated by its users, who also identified modifications that would improve the usability of future versions of the app.

## Abbreviations

AT: active transportation
mHealth: mobile health
PA: physical activity
SCAMPI: Smart City Active Mobile Phone Intervention
